# Insights Into circRNAs: Functional Roles in Lung Cancer Management and the Potential Mechanisms

**DOI:** 10.3389/fcell.2021.636913

**Published:** 2021-02-09

**Authors:** Bing Feng, Hao Zhou, Ting Wang, Xinrong Lin, Yongting Lai, Xiaoyuan Chu, Rui Wang

**Affiliations:** ^1^Department of Medical Oncology, Jinling Hospital, School of Medicine, Nanjing University, Nanjing, China; ^2^Department of Medical Oncology, Jinling Hospital, Nanjing Medical University, Nanjing, China; ^3^Department of Medical Oncology, Nanjing School of Clinical Medicine, Jinling Hospital, Southern Medical University, Nanjing, China

**Keywords:** circular RNAs (circRNAs), microRNAs (miRNAs), lung cancer, biomarker, prognosis

## Abstract

Lung cancer is the most prevalent cancer globally. It is also the leading cause of cancer-related death because of the late diagnosis and the frequent resistance to therapeutics. Therefore, it is impending to identify novel biomarkers and effective therapeutic targets to improve the clinical outcomes. Identified as a new class of RNAs, circular RNAs (circRNAs) derive from pre-mRNA back splicing with considerable stability and conservation. Accumulating research reveal that circRNAs can function as microRNA (miRNA) sponges, regulators of gene transcription and alternative splicing, as well as interact with RNA-binding proteins (RBPs), or even be translated into proteins directly. Currently, a large body of circRNAs have been demonstrated differentially expressed in physiological and pathological processes including cancer. In lung cancer, circRNAs play multiple roles in carcinogenesis, development, and response to different therapies, indicating their potential as diagnostic and prognostic biomarkers as well as novel therapeutics. In this review, we summarize the multi-faceted functions of circRNAs in lung cancer and the underlying mechanisms, together with the possible future of these discoveries in clinical application.

## Introduction

Nowadays, lung cancer is the most frequent cause of cancer-related mortality (Siegel et al., 2017). Non-small cell lung cancer (NSCLC), including lung adenocarcinoma, squamous cell lung carcinoma, and large cell lung carcinoma, constitutes about 85% of lung cancer cases. Although the novel therapeutic strategies such as targeting drugs toward the epidermal growth factor receptor (EGFR), anaplastic lymphoma kinase (ALK), and immune checkpoints programmed cell death-1 (PD-1) and/or programmed cell death-1 ligand (PD-L1) have led to an great progress of advanced NSCLC patients among the past decades, the long-term survival of lung cancer remains unfavorable because of the late diagnosis and the frequent resistance to therapeutics (Chansky et al., 2017; Kris et al., 2017). Therefore, the identification of sensitive biomarkers for early detection and prognosis estimation, as well as effective therapeutic targets is urgently needed to improve the clinical outcomes.

Circular RNAs (circRNAs) are identified as a new class of endogenous RNAs derived from back splicing. Lacking the 3′-poly(A) tails and 5′-end caps (Suzuki and Tsukahara, 2014), circRNAs have closed loop structures generated from the ligation of exons, introns, or both (Wang et al., 2019b), thus are divided into the three main subtypes as exonic circRNAs (EcircRNAs), intronic circRNAs (ciRNAs), and exonic circRNAs with introns (EIciRNAs), respectively. EcircRNAs exist in the eukaryotic cytoplasm, while ciRNAs and EIciRNAs are mainly in the nucleus. Other subtypes of circRNAs include intergenic circRNAs, tRNA intronic circRNAs (tricRNAs), antisense circRNAs, overlapping circRNAs, circRNA rRNAs (circrRNAs), and intragenic circRNAs (Liang et al., 2020). Owing to the absence of the 3′ and 5′ ends, circRNAs exhibit much more stability and conservation than the linear RNAs and are insusceptible to RNA exonuclease or RNase R-induced degradation. In general, circRNAs are expressed at lower levels than the host genes (Enuka et al., 2016). Although discovered half a century ago (Sanger et al., 1976), circRNAs are recently considered as the by-products from pre-mRNA back splicing without important biological functions. Currently, a large body of circRNAs have been demonstrated differentially expressed in physiological and pathological states including cancer due to the development of next-generation sequencing and bioinformatic technologies (Dragomir and Calin, 2018). In lung cancer, circRNAs reveal multiple roles in carcinogenesis, development, and response to therapies, implying their potential roles as not only the diagnostic and prognostic biomarkers but also novel therapeutics.

## Biogenesis of circRNAs

RNA alternative splicing is a basic gene expression event in eukaryotic cells. Unlike conventional splicing of mRNA, circRNAs are mainly produced from back-splicing process by ligating a downstream 5′ site with an upstream 3′ site and forming a single-strand closed loop (Jeck et al., 2013). After that, all or part of introns will be removed by the spliceosome and the rest of sequences are to be connected, generating the corresponding subtypes of circRNAs. Exon-skipping is another mechanism for circRNA circularization. It is reported that exon-skipping promotes the shaping process of the spliced lariat containing the circularized exon (Kelly et al., 2015). Furthermore, RNA-binding proteins (RBPs) are demonstrated to be able to induce circRNAs formation. For example, splicing factor muscleblind (MBL) has binding sites on flanking introns of its pre-mRNA and is able to bring the two splicing sites close together and facilitate circularization (Ashwal-Fluss et al., 2014). Enzyme adenosine deaminase 1 (ADAR1) can inhibit circRNAs’ expression by Adenosine-to-Inosine editing to diminish RNA pairing structure of flanking introns and diminish the back-splicing efficiency (Ivanov et al., 2015). RNA-binding protein quaking (QKI) is also proved to regulate circRNAs’ biogenesis by binding to sites flanking circRNAs forming exons to induce exon circularization during epithelial–mesenchymal transition (EMT) (Conn et al., 2015).

## Functions and Mechanisms of circRNAs

**FIGURE 1:**
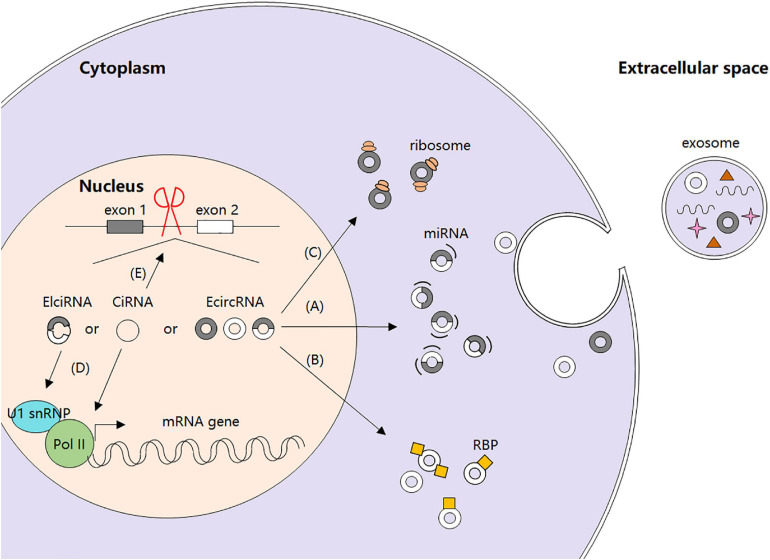
Biogenesis and regulatory functions of circRNAs. **(A)** circRNAs as miRNA sponges. **(B)** circRNA-protein interactions. **(C)** circRNAs and protein translation. **(D)** circRNAs and gene transcription. **(E)** circRNAs and alternative splicing.

Recently, increasing studies have focused on circRNAs’ biological functions and their regulation. It is confirmed that circRNAs can function as microRNA (miRNA) sponges to stop miRNAs from regulating gene expression via a circRNA–miRNA–mRNA pathway (Hansen et al., 2013). Moreover, circRNAs can act as regulators of gene transcription and alternative splicing, as well as interact with RBPs, or even be translated into peptides or full-length proteins directly (Liu et al., 2017) (Figure 1).

### circRNAs as miRNA Sponges

miRNAs are small non-coding RNAs that post-transcriptionally regulate gene expression by base paring with specific mRNA target sequences, thereby leading to translational inhibition or mRNA degradation (Salmanidis et al., 2014). They can be endogenously sponged by long non-coding RNAs (lncRNAs) owing to the presence of the miRNA response element (MRE) in lncRNA sequences (Cesana et al., 2011). It has been shown that some circRNAs located in cytoplasm also have complementary binding sites of miRNAs and can thus function as competing endogenous RNAs (ceRNAs) to compete with miRNAs and further regulate cellular functions (Zhong et al., 2018). For instance, EcircRNA ciRS-7 is reported to harbor more than 60 conserved miRNA seed match segments for miR-7, thereby antagonizing miR-7 biological activity and functions (Hansen et al., 2013). Subsequent studies confirm the importance of ciRS-7 as miR-7 sponges in many pathological processes including myocardial infarction, insulin secretion, and carcinogenesis (Pan et al., 2018).

### circRNA–Protein Interactions

**FIGURE 2:**
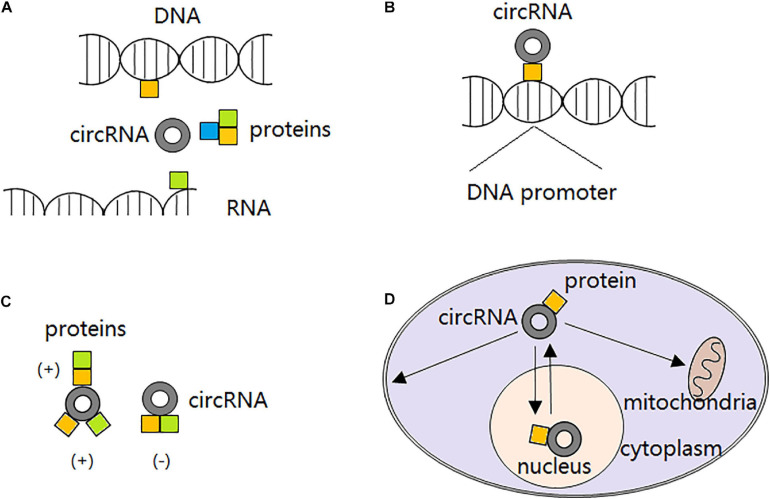
circRNA-protein interactions. **(A)** circRNAs block proteins from interacting with DNAs, RNAs or other proteins, thereby compromising their original functions. **(B)** circRNAs recruit transcription factors, chromatin remodelers, and DNA or histone modifying enzymes to the promoters, altering transcription either positively or negatively. **(C)** circRNAs strengthen interactions between proteins through direct binding to both of them or just one of them; or dissociate interactions between proteins originally combined together by direct binding to both of them. **(D)** circRNAs transport proteins between nucleus and cytoplasm, and to the mitochondria or membrane.

Recently, it is demonstrated that certain circRNAs can serve as protein decoys through binding to RBPs and regulate their interaction with DNAs, RNAs, and/or other proteins. For example, with several MBL binding sites, circMbl is able to sponge out the excessive MBL proteins and maintain its expression balance (Ashwal-Fluss et al., 2014). circANRIL can bind to pescadillo homolog 1 (PES1) and restrain exonuclease-mediated pre-rRNA (ribosomal RNA) processing (Holdt et al., 2016). circPABPN1 binds to HuR and prevents its binding to PABPN1 mRNA, resulting in PABPN1 translation attenuation (Abdelmohsen et al., 2017). circ-Foxo3 participates in the composition of a ternary complex by binding to cyclin-dependent kinase 2 (CDK2) and cyclin-dependent kinase inhibitor 1 (CDKN1), leading to impaired function of CDK2 and cell cycle arrest (Du et al., 2016). However, not all the circRNAs interacting with RBPs inhibit proteins functions. Particularly, circ-Amotl1 is reported to interact with and stabilize oncogene *c*-myc and upregulate *c*-myc targets, thereby promoting tumorigenesis (Yang Q. et al., 2017). The second action mode of circRNAs in interacting with proteins is to serve as protein recruiters. They can recruit not only transcription factors (Wang et al., 2019c), but also chromatin remodelers (Ding et al., 2019) and DNA or histone modifying enzymes (Chen N. et al., 2018) to the promoters and alter transcription either positively or negatively. Furthermore, circRNAs are able to alter interactions between proteins. In detail, circRNAs can strengthen interactions between proteins through direct binding to both of them (Huang S. et al., 2019) or just one of them (Fang et al., 2018), or dissociate interactions between proteins originally combined together by direct binding to both of them (Fang et al., 2019). Interestingly, it is reported that some circRNAs can also serve as protein transporters. circRNAs transport proteins between nucleus and cytoplasm (Yang Z. et al., 2017; Wang et al., 2019e), and to the mitochondria (Liu et al., 2020) or membrane (Du et al., 2020) (Figure 2).

### circRNAs and Protein Translation

Although initially considered as non-coding RNAs (Jeck et al., 2013), circRNAs are shown to be translatable in human transcriptome *in vivo* by ribosome footprinting and mass spectrometry analysis recently (Pamudurti et al., 2017). With the help of internal ribosome entry site (IRES) which is a non-circle structure, circRNA can be translated into either small peptides or full-length proteins. For example, circ-SHPRH is reported to encode the protein SHPRH-146aa, protect full-length SHPRH from degradation by the ubiquitin proteasome, and ultimately inhibit glioma tumorigenesis (Zhang et al., 2018a). An endogenous circFBXW7 can be encoded into a 21 kDa protein with suppressive roles in malignant phenotypes of human glioblastoma (Yang et al., 2018). Moreover, extensive *N6*-methyladenosine modification can drive the cap-independent translation of circRNAs together with m6A reader YTHDF3 and translation initiation factors eIF3A and eIF4G2 (Yang Y. et al., 2017).

Known circRNAs and their protein production are listed in Table 1.

**TABLE 1:** circRNAs and protein translation.

circRNA	Peptide/protein	References
circSHPRH	SHPRH-146aa	Zhang et al., 2018a
circFBXW7	FBXW7-185aa	Yang et al., 2018
HPV16 circE7	E7 oncoprotein	Zhao et al., 2019
circβ-catenin	β-catenin-370aa	Liang et al., 2019
circAKT3	AKT3-174aa	Xia et al., 2019
circPPP1R12A	PPP1R12A-73aa	Zheng et al., 2019
circPIN-Texon2	PINT87aa	Zhang et al., 2018b
circGprc5a	circGprc5a-peptide	Gu et al., 2018
circLgr4	circLgr4-peptide	Zhi et al., 2019

### circRNAs and Gene Transcription

It is demonstrated that certain ElciRNAs and ciRNAs may act as transcriptional regulators. For instance, ElciRNAs circEIF3J and circPAIP2 are reported to promote their parental genes promotion by a specific U1 small nuclear RNA (snRNA)-ElciRNA interaction. Mechanically, EIciRNAs bind to U1 small nuclear ribonucleoprotein (snRNP) through interaction with U1 snRNA and to form EIciRNA–U1 snRNP complexes, which will further interact with RNA polymerase II (Pol II) transcription complex of the parental gene and enhance gene transcription (Li et al., 2015). Besides, ciRNA ci-ankrd52 is able to accumulate to its transcription sites and positively regulate elongation Pol II machinery, suggesting a *cis*-regulatory role of ciRNAs in expression of their parental genes (Zhang et al., 2013).

### circRNAs and Alternative Splicing

RNA alternative splicing is a basic gene expression event in eukaryotic cells. During this process, the backsplicing of circRNAs competes with the linear splicing of pre-mRNAs for splicing sites. For example, derived from the second exon of splicing factor MBL, circMbl itself and its flanking introns have conserved MBL binding sites, indicating that MBL may have effects on alternative splicing and modulate the balance between the backsplicing of circRNAs and the linear splicing of pre-mRNAs (Ashwal-Fluss et al., 2014). Meanwhile, circRNAs can act as “mRNA traps” during the back-splicing process by sequestering the translation start site to prevent translation of certain normal linear transcripts and reduce the expression of the very proteins. For instance, EcircRNAs generated from Formin (Fmn) and Duchenne muscular dystrophies traps (DMD) genes are able to cause inactivation of RNA transcripts with certain deletion mutations, thereby diminishing the expression levels of the corresponding functional proteins (Chao et al., 1998; Gualandi et al., 2003).

## circRNAs and Lung Cancer

Emerging evidence suggests that circRNAs are abnormally expressed and playing endogenous regulatory roles in carcinogenesis and development of lung cancer, including cell viability, apoptosis, autophagy, invasion, migration, tumor microenvironment (TME) regulation (such as tumor immunosuppression, angiogenesis, hypoxia, and metabolic abnormalities), and therapeutic sensitivities. Moreover, circRNAs own relative specificity and stability compared to other non-coding RNAs, making them more attractive as novel diagnostic and prognostic biomarkers as well as promising therapeutics in lung cancer management. With the development of high-throughput sequencing technology, circRNA expressions in cell lines, tissue samples, and liquid biopsies (especially blood) from lung cancer patients have been detected (Wang et al., 2019a; Zhang et al., 2020). Functional validation assays as well as bioinformatic analysis have been performed to reveal the interaction network of circRNAs and other regulatory factors in lung cancer tumorigenesis and development.

### circRNAs as Diagnostic and Prognostic Biomarkers for Lung Cancer

With advantages such as non-invasion, specificity, reproducibility, and sensitivity, circRNAs can act as biomarkers for lung cancer pathological subtyping. For example, hsa_circ_0013958 is indicated to be used as a potential biomarker for screening and early detecting lung adenocarcinoma (Zhu et al., 2017). In a recent work, circRNA expressions are profiled in both lung adenocarcinoma and squamous cell carcinoma and the result indicates that the two subtypes exhibit distinct circRNA expression signatures (Wang et al., 2019a). Similarly, circNOL10 expression varies significantly between lung adenocarcinoma and lung squamous cell carcinoma and has close relationship with the degree of differentiation (Nan et al., 2018). Another study confirms that circ-STXBP5L is selectively expressed in small cell lung cancer (SCLC) samples compared with NSCLC (Zhang et al., 2020). These results highlight the important diagnostic value of circRNAs in pathological classification of lung cancer.

Moreover, mounting studies demonstrate that circRNAs can also serve as prognostic biomarkers for lung cancer occurrence, development, tumor node metastasis (TNM) staging, pathological grade, and lymphatic metastasis. Generally, many circRNAs are upregulated in lung cancer and are considered to be “onco-circRNAs” due to their positive roles in cancer cell proliferation, invasion, and migration, as well as the negative regulation on cancer cell apoptosis. Vice versa, circRNAs that are downregulated in lung cancer with tumor-suppressive functions are considered as “tumor-suppressive circRNAs.”

#### Onco-circRNAs in Lung Cancer

To date, a multitude of onco-circRNAs have been identified in lung cancer and proposed as potential biomarkers for prognosis.

As is well-known that metastasis is one of the main characteristics of malignant tumors including lung cancer. It is reported that friend leukemia virus integration 1 (FLI1) exonic circRNA FECR1 can promote SCLC metastasis by increasing rho-associated coiled-coil kinase 1 (ROCK1) expressions through direct inhibition of miR584-3p (Li L. et al., 2019). It is described above that circ-STXBP5L participates in the carcinogenesis of SCLC as an onco-circRNA by sponging miR-224-3p and miR-512-3p and regulating a subset of target genes, including Akts, NFκB and Pik3ca (Zhang et al., 2020). As for NSCLC, the data are more detailed. For instance, over-expressed in EGFR-resistant H1975 cells, circRNA CCDC66 is regulated by HGF/c-Met to increase EMT process and drug resistance of lung adenocarcinoma (Joseph et al., 2018). Produced from the EML4-ALK fusion, circRNA F-circEA-2a reveals positive effect on cell invasion and migration in NSCLC, highlighting its critical role in EML4-ALK-positive NSCLC (Tan et al., 2018). circRNA_102231 promotes cellular proliferation, invasion, and migration in lung cancer. Highly expressed circRNA_102231 may serve as a biomarker for both diagnosis and prognosis for lung cancer patients (Zong et al., 2018). It is demonstrated that circHIPK3 can restore lung cancer cell survival and proliferation via sponging miR-124 and regulating expression of its potential targets such as SphK1, STAT3, and CDK4 (Yu et al., 2018). Particularly, circHIPK3 also functions as a negative autophagy regulator in lung cancer through the miR124-3p-STAT3-PRKAA pathway which is dependent on STK11 status (Chen et al., 2020). circHIPK3 regulates the EMT progress of NSCLC through miR-149-mediated Forkhead Box transcription factor FOXM1 expression regulation, closely correlated with the aggressive potential and unfavorable prognosis (Lu et al., 2020). CircPVT1 positively regulates NSCLC cell proliferation, invasion, and metastasis by sponging miR-125b and activating the corresponding E2F2 signaling pathway (Li X. et al., 2018). It can also act as a competing endogenous RNA for miR-497 and indirectly increase the expression of Bcl-2, leading to promoted NSCLC progression and predicting poor survival of the patients (Qin et al., 2019).

Hypoxia caused by the instability of the tumor-associated microvasculature is one of the key reasons for cancer progression. It is reported that circ_0000376 can promote NSCLC progression by regulating the miR-1182/NOVA2 axis and is relative to the poor overall survival of NSCLC patients. Hypoxia enhances circ_0000376 expression and promotes the glycolysis, viability, invasion and migration of NSCLC cells. Manipulated inhibition of circ_0000376 suppresses the progressive activities of hypoxia-induced NSCLC cells both *in vitro* and *in vivo* (Li C. et al., 2020).

Several circRNAs are verified to effect both cell proliferation and apoptosis in lung cancer. For instance, upregulated hsa_circ_0000064 demonstrates effect on cell proliferation, metastasis, and apoptosis by regulating target genes such as cell cycle regulators p21WAF1, cyclin D1, and CDK6, as well as apoptotic factors caspase-3, caspase-9, and bax (Luo et al., 2017). Serving as a sponge for miR-503, circ-BANP promotes proliferation, invasion, and migration while attenuates apoptosis of lung cancer cells through promotion of LARP1 expression (Han et al., 2018). circ-FOXM1 works as a ceRNA to target PPDPF and MACC1 by sponging miR-1304-5p in NSCLC. It promotes cellular proliferation, invasion, and migration, and suppresses apoptosis, thus playing oncogenic roles in progression of NSCLC. The elevation of circ-FOXM1 in NSCLC is proved to be strongly linked to advanced TNM stages, lymph node invasion, as well as dismal prognosis (Liu G. et al., 2019). Commonly upregulated in NSCLC tissues and cell lines, circ_0026134 facilitates NSCLC proliferation and metastatic properties and weakens cell apoptosis, dependent on the sponge and down-regulation of miR-1287 and miR-1256 (Chang et al., 2019). Similarly, circRNA 100146 is highly expressed and playing an oncogenic role in the progression of NSCLC. It enhances NSCLC cell proliferation and invasion and inhibits cell apoptosis through direct binding to miR-361-3p and miR-615-5p and affections on multiple downstream molecules such as NFAT5, COL1A1, TRAF3, and MEF2C (Chen L. et al., 2019).

It is shown that lung cancer progression is also promoted by increased glycolysis. For example, Enolase 1 (ENO1) is a glycolysis enzyme which performs crucial roles in glucose metabolism and contributes to progression of lung cancer. Recently, circ-ENO1 and its host gene ENO1 are reported to be upregulated in lung adenocarcinoma. Mechanistically, circ-ENO1 acts as a ceRNA of miR-22-3p and upregulates ENO1 expression, promoting glycolysis and tumor progression in lung adenocarcinoma. Silencing of circ-ENO1 inhibits glycolysis, cell proliferation, migration and EMT of lung adenocarcinoma (Zhou J. et al., 2019).

Tumor immune microenvironment is another pivotal factor for the development of lung cancer where cancer cells interact with immune cells to facilitate immune evasion. For example, NSCLC-derived intracellular and extracellular PD-L1 can not only promote cancer progression and drug resistance but also facilitate tumor immune evasion (Li Y. et al., 2019). It is recently demonstrated that circ-CPA4 is high-expressed in NSCLC and can regulate cell growth, metastasis, stemness and drug resistance as well as inactivate CD8+ T cells in tumor immune microenvironment through miRNA let-7/PD-L1 regulatory axis. Inhibition of circ-CPA4 suppresses NSCLC cell growth, mobility and EMT, while enhances cell death via downregulation of let-7/PD-L1 axis. Furthermore, circ-CPA4 can positively regulate the expression of exosomal PD-L1 which promotes NSCLC cell stemness and increases the resistance toward cisplatin (Hong et al., 2020).

Dysregulated onco-circRNAs in lung cancer, their functions, and the underlying mechanisms are listed in Table 2.

**TABLE 2:** Dysregulated onco-circRNAs in lung cancer.

circRNA	Function	Mechanism	References
circFECR1	Metastasis of SCLC	miR584-3p/ROCK1	Li L. et al., 2019
circSTXBP5L	Carcinogenesis of SCLC	miR-224-3p and miR-512-3p/Akts, NFκB, and Pik3ca	Zhang et al., 2020
circ_100876	Prognosis values	?	Yao et al., 2017
circCCDC66	EMT and drug resistance of lung adenocarcinoma	?	Joseph et al., 2018
circF-circEA-2a	Invasion and migration of NSCLC	?	Tan et al., 2018
circRNA_102231	Proliferation, invasion, and migration of lung cancer	?	Zong et al., 2018
hsa_circRNA_103809	Proliferation and invasion of lung cancer	miR-4302/ZNF121/MYC	Liu W. et al., 2018
circHIPK3	Cell survival and proliferation of lung cancer	miR-124/SphK1, STAT3, and CDK4	Yu et al., 2018
	Autophagy of lung cancer	miR124-3p/STAT3/PRKAA	Chen et al., 2020
	EMT and aggressiveness of NSCLC	miR-149/FOXM1	Lu et al., 2020
circPVT1	Proliferation, invasion, and metastasis of NSCLC	miR-125b/E2F2	Li X. et al., 2018
	NSCLC progression	miR-497/Bcl-2	Qin et al., 2019
circMAN2B2	Proliferation and invasion of lung cancer	miR-1275/FOXK1	Ma et al., 2018
circ_0067934	Tumorigenesis, EMT, and metastasis of NSCLC	?	Wang and Li, 2018
hsa_circ_0020123	Proliferation, invasion, and migration of NSCLC	miR-144/ZEB1 and EZH2	Qu et al., 2018
	Growth, invasion, migration, and apoptosis of NSCLC	miR-488e3p/ADAM9	Wan et al., 2019
circFADS2	Proliferation and invasion of lung cancer	miR-498/FOXO1/KLF6	Zhao et al., 2018
circ_0016760	Progression of NSCLC	miR-1287/GAGE1	Li Y. et al., 2018
circPRKCI	Proliferation and tumorigenesis of lung adenocarcinoma	miR-545 and miR-589/E2F7	Qiu et al., 2018
circCMPK1	Cell proliferation of NSCLC	miR-302e/cyclin D1	Cui et al., 2020
circFGFR1	Proliferation, migration, invasion, and immune evasion abilities of NSCLC	miR-381-3p/CXCR4	Zhang P. et al., 2019
circP4HB	EMT and metastasis of NSCLC	miR-133a-5p/vimentin	Wang et al., 2019f
hsa_circ_000984	Proliferation and metastasis of NSCLC	Wnt/β-catenin signaling	Li X. et al., 2019
circ_0003645	NSCLC progression	miR-1179/TMEM14A	An et al., 2019
circZFR	NSCLC progression	miR-101-3p/CUL4B	Zhang H. et al., 2019
hsa_circ_0023404	Proliferation, invasion, and migration of NSCLC	miR-217/ZEB1	Liu C. et al., 2019
F-circSR1 and 2	Migration and invasion of NSCLC	?	Wu et al., 2019
circPRMT5	NSCLC progression	miR-377, miR-382, and miR-498/EZH2	Wang et al., 2019h
circPIP5K1A	Proliferation and metastasis of NSCLC	miR-600/HIF-1α	Chi et al., 2019
circATXN7	Proliferation and invasion abilities of NSCLC	?	Huang Q. et al., 2019
circFGFR3	Proliferation and invasion of NSCLC	miR-22-3p/Gal-1, p-AKT and p-ERK1/2	Qiu et al., 2019
circ_0043278	Proliferation, invasion and migration of NSCLC	miR-520f/ROCK1, CDKN1B and AKT3	Cui et al., 2019
circRAD23B	Progression of NSCLC	miR-593e3p/CCND2 and miR-653e5p/TIAM1	Han et al., 2019
circNT5E	Cell growth, proliferation, and migration of NSCLC	miR-134	Dong et al., 2020
hsa_circ_0013958	Proliferation, invasion, and apoptosis of lung adenocarcinoma	miR-134/cyclin D1	Zhu et al., 2017
circ_0000376	Glycolysis, cell viability, invasion and migration of NSCLC	miR-1182/NOVA2	Li Y. et al., 2020
hsa_circ_0000064	Proliferation, apoptosis, and metastasis of lung cancer	Cell cycle regulators (p21WAF1, cyclin D1, and CDK6) and apoptotic factors (caspase-3, caspase-9, and bax)	Luo et al., 2017
hsa_circ_0007385	NSCLC progression	miR-181/Bcl-2 and CDK1	Jiang et al., 2018
circBANP	Proliferation, invasion, migration, and apoptosis of lung cancer	miR-503/LARP1	Han et al., 2018
circVANGL1	Proliferation, migration, invasion, and apoptosis of NSCLC	miR-195/Bcl-2 and Bax	Wang et al., 2019d
circFOXM1	Proliferation, invasion, migration, and apoptosis of NSCLC	miR-1304-5p/PPDPF and MACC1	Liu G. et al., 2019
circ_0026134	Proliferation, metastasis, and apoptosis of NSCLC	miR-1287/PIK3R3 and miR-1256/TCTN1	Chang et al., 2019
circRNA 100146	Proliferation, invasion, and apoptosis of NSCLC	miR-361-3p and miR-615-5p/NFAT5, COL1A1, TRAF3, and MEF2C	Chen L. et al., 2019
circCDR1	Cell viability, migration, invasion, and apoptosis of NSCLC	miR-219a-5p/SOX5	Li Y. et al., 2020
circ-ENO1	Glycolysis, proliferation, apoptosis, migration and EMT of lung adenocarcinoma	miR-22-3p/ENO1	Zhou J. et al., 2019
circ-CPA4	Cell growth, mobility, stemness, drug resistance, and CD8+ T cell inactivation in the tumor immune microenvironment of NSCLC	let-7/PD-L1	Hong et al., 2020

#### Tumor-Suppressive circRNAs in Lung Cancer

A series of circRNAs have been found downregulated in lung cancer and thus considered as tumor suppressors. For instance, downregulated in SCLC and chemo-resistant NSCLC cells, circRNA cESRP1 enhances drug sensitivity by directly binding to and repressing miR-93-5p, thereby up-regulating the expression of Smad7 and p21, forming a negative feedback loop to regulate EMT process dependent of transforming growth factor-β (TGF-β) (Huang W. et al., 2019).

In NSCLC, circRNA ITCH is reported markedly decreased in cancer tissues with negative regulation on the proliferation of cancer cells by down-regulating oncogenic miR-7 and miR-214 as well as up-regulating T-cell factor, β-catenin, c-Myc, and cyclin D1, thereby enhancing the activation of the Wnt/β-catenin signaling pathway (Wan et al., 2016). circ_0001649 is demonstrated to have a decreased expression in NSCLC tissues and cell lines with suppressive functions on cell growth and metastasis both *in vitro* and *in vivo* partially by sponging out miR-331-3p and miR-338-5p. The down-regulation of circ_0001649 is highly interrelated with advanced TNM stage, positive lymph node metastasis, and poor prognosis of NSCLC patients (Liu T. et al., 2018). circPTK2 promotes TIF1γ expression and inhibits TGF-β-induced EMT and metastasis in NSCLC dependent on miR-429/miR-200b-3p sponging (Wang et al., 2018). circRNA-FOXO3 expression is also found decreased in NSCLC and correlated with clinical outcomes. circRNA-FOXO3 inhibits NSCLC cell proliferation, invasion, and migration by acting as a ceRNA to sponge miR-155 and release FOXO3 expression (Zhang et al., 2018c).

Cell apoptosis is also regulated by certain tumor-suppressive circRNAs in lung cancer. For example, acting as an endogenous sponge for miR-1252, has_circ_0043256 can upregulate the expression of ITCH and finally inhibit Wnt/β-catenin pathway, leading to suppression of cell proliferation and enhancement of apoptosis in NSCLC (Tian et al., 2017). circNOL10 is downregulated in both lung cancer tissues and cells and conducive in differentiating lung adenocarcinoma and lung squamous cell carcinoma. Furthermore, circNOL10 inhibits lung cancer by enhancing transcriptional regulation of the HN polypeptide family, which exerts pivotal functions on biological processes such as proliferation, apoptosis, and cell cycle progression (Nan et al., 2018).

Dysregulated tumor-suppressive circRNAs in lung cancer, their functions, and the underlying mechanisms are listed in Table 3.

**TABLE 3:** Dysregulated tumor-suppressive circRNAs in lung cancer.

circRNA	Function	Mechanism	References
circESRP1	EMT and drug sensitivity of SCLC	miR-93-5p/Smad7/p21(CDKN1A)/TGF-β	Huang W. et al., 2019
circITCH	proliferation of NSCLC	miR-7 and miR-214/T-cell factor, β-catenin, c-Myc, and cyclin D1	Wan et al., 2016
circ_0001649	growth and metastasis of NSCLC	miR-331-3p and miR-338-5p	Liu T. et al., 2018
circPTK2	EMT and metastasis of NSCLC	miR-429 and miR-200b-3p/TIF1γ/TGF-β	Wang et al., 2018
circFOXO3	Proliferation, invasion, and migration of NSCLC	miR-155/FOXO3	Zhang et al., 2018c
hsa_circ_100395	Proliferation, invasion, and migration of lung cancer	miR-1228/TCF21	Chen D. et al., 2018
hsa_circ_0033155	Proliferation and migration of NSCLC	PTEN	Gu et al., 2019
circSMARCA5	Proliferation and chemo-sensitivity of NSCLC	miR-19b-3p/HOXA9	Wang et al., 2019g; Tong, 2020
hsa_circ_0007059	Proliferation and EMT of lung cancer	miR-378/Wnt/β-catenin and ERK1/2	Gao et al., 2019
circARHGAP10	NSCLC progression	miR-150-5p/GLUT1	Jin et al., 2019
circPTPRA	EMT and metastasis of NSCLC	miR-96-5p/RASSF8	Wei et al., 2019
has_circ_0043256	Proliferation and apoptosis of NSCLC	miR-1252/ITCH/Wnt/β-catenin	Tian et al., 2017
circNOL10	Proliferation, apoptosis, and cell cycle progression of lung cancer	SCLM1/the HN polypeptide family	Nan et al., 2018

### circRNAs and the Therapeutic Response of Lung Cancer

Except for chemotherapy, targeted therapies and immunotherapies have revolutionized the lung cancer management within the last decades. However, drug-resistance still develops after the treatment. Considering the multiple roles of circRNAs in lung cancer progression, it is not surprising to apply them as predictive biomarkers for the follow-up of patients. Furthermore, the detection of circRNAs in liquid biopsies has provided a more convenient method for the management of post-treatment follow-up.

#### Chemotherapy

With the assistance of a high-throughput circRNA microarray, a significant upregulation of 2,909 circRNAs as well as downregulation of 8,372 circRNAs are discovered in taxol-resistant A549 lung adenocarcinoma cells compared with the parental cells (Xu et al., 2018). Functional validation assays highlight the circRNA/miRNA networks in this context. The most pronouncedly enriched pathways for aberrant circRNA-related host genes include VEGFR, EGFR, integrin, and rho GTPase signaling, which are all involved in the progression of chemo-resistance.

Eukaryotic initiation factor 3 (EIF3) is one of the largest translation initiation factors. Previous studies have suggested the involvement of EIF3a in tumorigenesis and drug resistance of lung cancer. Recently, it is found that the expression of hsa_circ_0004350 and hsa_circ_0092857, both derived from EIF3a, varies prominently in cisplatin-resistant lung cancer cell line and the parental cell line. Manipulated regulation of the two circEIF3as affects cisplatin sensitivity of lung cancer cells. Further bioinformatic analysis indicates that the two circEIF3as are not only related to translational regulation, but also showing functional synergy with their parental gene EIF3a, thus serving as potential therapeutic targets during lung cancer management (Huang M.S. et al., 2019).

#### Targeted Therapy

EGFR-tyrosine kinase inhibitors (EGFR-TKIs) have become important constituents for NSCLC treatment these years. Despite the good initial responses, tumor progression is systematically observed due to the emergence of acquire resistance. Furthermore, detection for EGFR driver mutation is hindered by problems such as cancer heterogeneity and lack of cancer tissues. By screening circRNAs expression profile via circRNA microarray, it is found that hsa_circ_0109320 and hsa_circ_0134501 are upregulated in gefitinib-effective NSCLC patients. Moreover, hsa_circ_0109320 expression is associated with better progression-free survival (PFS), indicating its potential role as a prognostic biomarker for gefitinib-treated NSCLC patients (Liu Y. et al., 2019). Similarly, hsa_circ_0004015 is identified to be highly expressed in NSCLC cells and tissues. Patients with high expression of hsa_circ_0004015 often have a worse overall survival rate. Further study indicates that hsa_circ_0004015 promotes NSCLC progression and gefitinib resistance through sponge for miR-1183 and induction of 3-phosphoinositide-dependent protein kinase 1 (PDPK) as well as the downstream AKT pathway (Zhou Y. et al., 2019). Recently, circRNA expression profiles have been explored in Osimertinib (AZD9291)-resistant NSCLC cells and the result shows that the most modulated circRNAs are involved in regulation of cancer-related pathways including proliferation, invasion, apoptosis, and resistance to chemotherapeutic drugs as well as γ-radiation (Chen T. et al., 2019).

Another role of circRNAs during tumorigenesis is the formation of fusion circRNAs (f-circRNAs) derived from chromosomal translocations (Guarnerio et al., 2016). It is demonstrated that f-circEA-2a which derived from back splicing of the EML4-ALK fusion gene promotes cell invasion and migration but not cell proliferation in NSCLC. Interestingly, f-circEA-2a is detected in tumor tissues but not plasma of EML4-ALK-positive NSCLC patients (Tan et al., 2018).

#### Immunotherapy

Anti-PD-1-based immunotherapy has led to an effective response in multiple advanced cancers, lung cancer included. However, more than half of NSCLC patients lack a long-term response to this immunotherapy (Melosky et al., 2019). Emerging evidence show that dysregulated chemokine receptor expression is one of the critical intrinsic reasons for tumor-promotion and immune system evasion (Adrover et al., 2019). Recently, it is found that circFGFR1 may act as a sponge for miR-381-3p, thereby promoting NSCLC progression and resistance to anti-PD-1 therapy by up-regulating CXCR4 expression. CircFGFR1 is upregulated in NSCLC tissues with its expression closely correlated with unfavorable prognosis of NSCLC patients. Manipulated upregulation of circFGFR1 promotes the proliferation, invasion, migration, and immune evasion of NSCLC cells, while knockdown of CXCR4 resensitizes NSCLC cells to anti-PD-1 immunotherapy (Zhang P. et al., 2019). As is mentioned above, circ-CPA4 can regulate cell growth, mobility, stemness and drug resistance in NSCLC cells and inactivates CD8+ T cells in the tumor immune microenvironment through the let-7 miRNA/PD-L1 axis. On the one hand, PD-L1 selfregulates NSCLC cell malignant activities. On the other hand, secreted PD-L1 by exosomes inactivates CD8+ T cells by activating extracellular and intracellular pathways and mediates cell death to facilitate immune evasion (Hong et al., 2020).

### circRNAs as Promising Therapeutics for Lung Cancer

Regarding the onco- and tumor suppressive- roles in lung cancer, circRNAs provide insight into the exploration of novel strategies in lung cancer management. Moreover, as mentioned above, circRNAs are more suitable for targeted molecular therapy because of their stable, tissue specific, and ceRNA-equivalent characteristics. Further investigation is needed to translate circRNAs into clinics and provide a foundation for developing novel potential therapeutic strategies for lung cancer and improve the prognosis of the patients.

## Discussion

Despite significant advances in diagnosis and treatment, lung cancer remains the leading cause of death worldwide with its underlying mechanisms remaining largely undiscovered. A major obstacle of improving clinical outcomes is to identify sensitive biomarkers and novel therapeutics for individualized diagnosis and treatment of lung cancer. In recent years, thousands of circRNAs have been identified with the rapid development of NGS technology and bioinformatics. Although considered as splicing by-products initially, circRNAs are now becoming a hotspot in the field of cancer owing to their conservation across species, the relative high stability and abundance, and the accessibility in body fluids, especially blood. They reveal diverse regulatory functions on genes and proteins involved in cancer cell proliferation, invasion, migration, cell cycle, apoptosis, and drug sensitivity. Moreover, recent studies have shown that the role of circRNAs in cancer is mainly dependent on the circRNA–miRNA–mRNA regulatory network, indicating their potential functions in the regulation of transcriptional and post-transcriptional levels. Other mechanisms include interacting with RBPs, translating into either peptides or full-length proteins, and regulating transcription. This provides novel biomarkers for lung cancer prognosis prediction, especially considering the lack of reliable clinical biomarkers in SCLC. In addition, engineered circRNAs can be applied to effectively sequester not only RNAs (including miRNAs), but also DNAs and RBPs with specific sequences both *in vitro* and *in vivo*, providing promising molecular targets for the therapy of lung cancer (Lasda and Parker, 2014).

However, it should be noted that the study on circRNAs in lung cancer is still in the early stage. The functions and underlying mechanisms of circRNAs in the regulatory network of tumorigenesis and progression remains largely unknown and needs to be further studied in more cell lines and clinical samples. Moreover, problems such as the high cost of experiment, difficulties in detection and monitoring, and the potential side effects, are still limiting the application of circRNAs in clinic. We hope that in the future, with the rapid development of bioinformatic technology and universal studies in patient tissue samples, circRNAs could help to achieve better individualized diagnosis and treatment of lung cancer.

## Author Contributions

RW conceptualized the review. BF and HZ were the major contributors in writing the manuscript. TW designed the figures. XL and YL prepared the tables. XC critically reviewed and edited the manuscript. All the authors read and approved the final manuscript.

## Conflict of Interest

The authors declare that the research was conducted in the absence of any commercial or financial relationships that could be construed as a potential conflict of interest.

## Abbreviations

ADAR1: adenosine deaminase 1
CDK2: cyclin-dependent kinase 2
CDKN1: cyclin-dependent kinase inhibitor 1
ceRNA: competing endogenous RNA
circRNAs: circular RNAs
circrRNAs: circRNA rRNAs
ciRNAs: intronic circRNAs
CXCR4: C-X -C motif chemokine receptor 4
EcircRNAs: exonic circRNAs
EGF: epidermal growth factor
EGFR: epidermal growth factor receptor
EIF3: eukaryotic initiation factor 3
ElciRNAs: exonic circRNAs with introns
EMT: epithelial-to-mesenchymal transition
f-circRNAs: fusion circRNAs
FLI1: friend leukemia virus integration 1
FOXM1: Forkhead Box (Fox) transcription factor 1
HIF: hypoxia-inducible factor
IRES: internal ribosome entry site
lncRNA: long non-coding RNA
m6A: *N6*-methyladenosine
MBL: splicing factor muscleblind
miRNA: microRNA
NSCLC: non-small cell lung cancer
ORF: open reading frame
PD-1: programmed cell death-1
PD-L1: programmed cell death-1 ligand
PDPK: phosphoinositide-dependent protein kinase
PES1: pescadillo homolog 1
PFS: progression-free survival
Pol II: RNA polymerase II
PTEN: phosphatase and tensin homolog deleted on chromosome 10
QKI: RNA-binding protein quaking
RBP: RNA-binding protein
ROCK1: Rho-associated coiled-coil kinase 1
rRNA: ribosomal RNA
SCLC: small cell lung cancer
snRNA: small nuclear RNA
snRNP: small nuclear ribonucleoprotein
TGF- β: transforming growth factor- β
TKI: tyrosine kinase inhibitor
TME: tumor microenvironment
TNM: tumor node metastasis
tricRNAs: tRNA intronic circRNAs.
